# Angioscopy-Guided Selective Pulmonary Thrombectomy and Angioscopy-Monitored Systemic Thrombosis for a Pulmonary Embolism

**DOI:** 10.7759/cureus.38365

**Published:** 2023-05-01

**Authors:** Sei Komatsu, Satoru Takahashi, Chikao Yutani, Tomoki Ohara, Mitsuhiko Takewa, Kazuhisa Kodama

**Affiliations:** 1 Cardiovascular Center, Osaka Gyoumeikan Hospital, Osaka, JPN

**Keywords:** thrombectomy, imaging, non-obstructive general angioscopy, thrombolysis, pulmonary embolism

## Abstract

Few modalities have the capacity to demonstrate massive or fragmented thrombi to evaluate the effect of catheter-based or systemic thrombosis for pulmonary embolism (PE). We herein present a patient who underwent a thrombectomy for PE using a non-obstructive general angioscopy (NOGA) system. Small floating mobile thrombi were aspirated using the original method, and massive thrombi were aspirated using the NOGA system. Systemic thrombosis was also monitored via NOGA for 30 minutes. Detachment of thrombi from the wall of the pulmonary artery began two minutes after infusion of a recombinant tissue plasminogen activator (rt-PA). Six minutes after thrombolysis, the thrombi lost their erythematous color, and the white thrombi gradually floated and dissolved. NOGA-guided selective pulmonary thrombectomy and NOGA-monitored systemic thrombosis contributed to improved patient survival. Rapid systemic thrombosis for PE using rt-PA was also demonstrated by NOGA.

## Introduction

Pulmonary embolism (PE) is a common cause of death due to cardiovascular disease [1]. The rate of recurrent PE while on anticoagulant medication is below 5% but can increase to 30% after a decade [2]. The incomplete dissolution of pulmonary blood clots results in persistent obstruction of the pulmonary arteries, resulting in chronic thromboembolic pulmonary hypertension [3,4]. Anticoagulation and systemic thrombolysis, catheter-directed thrombolysis, and surgery are the standard treatment options for PE [1]. Various hybrid techniques have been reported for catheter-directed thrombolysis [5]. A meta-analysis demonstrated that catheter-directed thrombolysis reduced all-cause mortality in PE cases compared to anticoagulation therapy. Moreover, all-cause mortality during hospitalization and the incidence of intracranial hemorrhage compared with those with systemic anticoagulation was reduced [6]. It is essential to confirm the effectiveness of treatment. The gradual resolution of PE over days or months is evaluated using computed tomography [7]. Excessive application of contrast agents increases the risk of renal dysfunction [8]. Non-obstructive general angioscopy (NOGA) is a device that explores the interior structure of vessels such as the aorta, coronary arteries, and pulmonary arteries [9-11]. Few invasive modalities can demonstrate massive or fragmented thrombi to evaluate the effect of catheter-based or systemic thrombosis for pulmonary embolism (PE). This study reports the application of NOGA-guided selective pulmonary thrombectomy and NOGA-monitored systemic thrombosis.

## Case presentation

**Figure 1:**
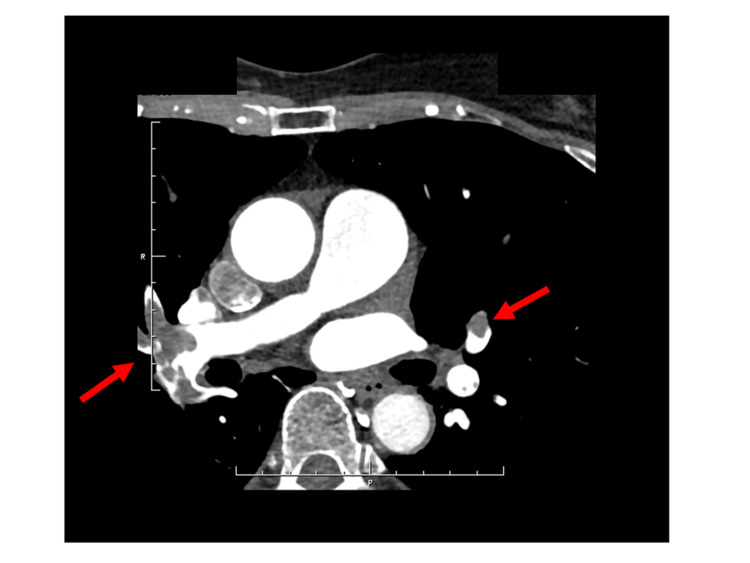
Axial computed tomography angiography of the pulmonary arteries with pulmonary thrombi on both sides Arrows indicate pulmonary thrombi on both sides of the pulmonary arteries.

A 71-year-old woman, complaining of exertional dyspnea for five days, was hospitalized. She was taking medication for depression. The patient had an oxygen saturation (SpO_2_) of 80% in the ambulance. Her heart rate was 100 bpm, and her respiratory rate was 24/min. Her blood pressure was 132/70 mmHg. Electrocardiography showed an S wave in lead I as well as Q waves and a negative T wave in lead III. Her arterial blood gas indicated respiratory failure (pH 7.467, partial pressure of oxygen (PO_2_) 68.3 mmHg, partial pressure of carbon dioxide (PCO_2_) 31.7 mmHg, bicarbonate (HCO_3_) 22.4 mEq/L, arterial oxygen saturation (SaO_2_) 94.8 %, and base excess (BE) -0.5 mEq/L) on room air. She had a D-dimer level of 33.20 μg/mL; her troponin T level was 28 pg/mL. The test for antiphospholipid antibodies gave negative results, and the protein C and S levels were normal. The transtricuspid pressure gradient was 17.9 mmHg by echocardiography. Computed tomography angiography of the pulmonary artery revealed submassive pulmonary thrombi on both sides of the pulmonary arteries (Figure 1).

**Figure 2:**
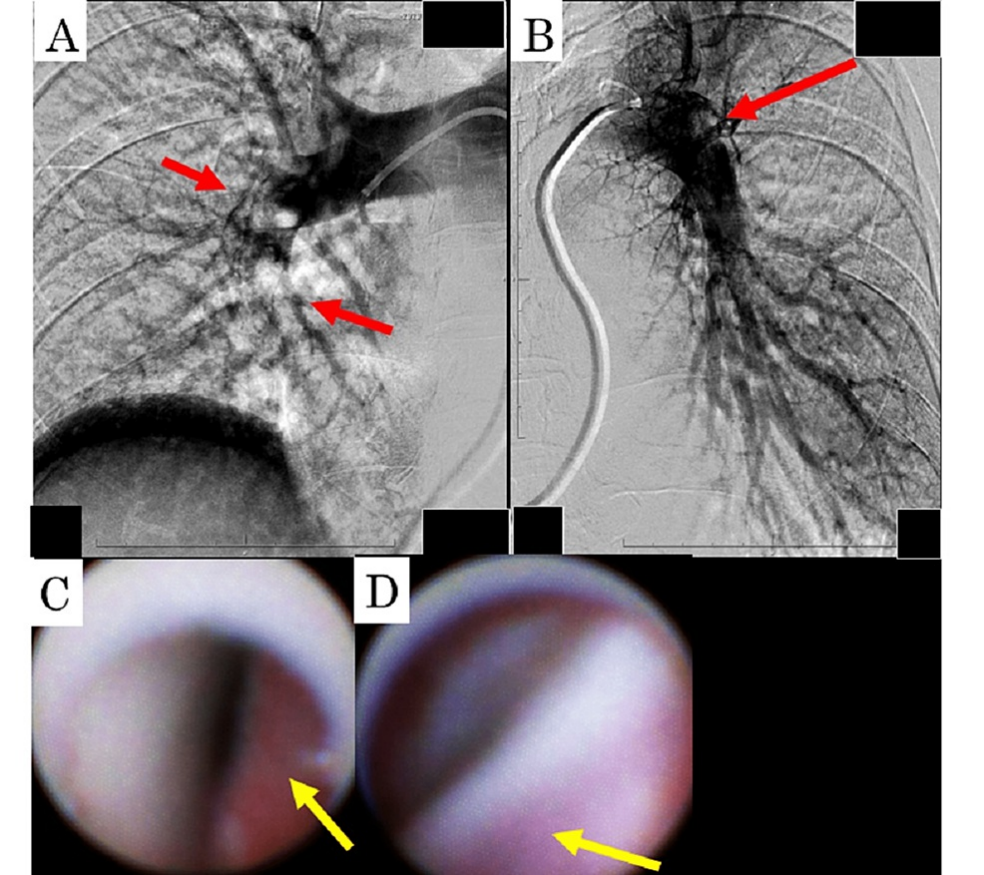
Invasive arteriography images and angioscopic images of the pulmonary arteries A. Invasive pulmonary arteriography of the right pulmonary arteries. Arrows indicate the detected thrombi. B. Invasive pulmonary arteriography of the left pulmonary arteries. Arrows indicate the detected thrombi. C. An angioscopic image of the A^6^ segment in the right pulmonary artery. An arrow indicates red surfaces with protruding white mixed thrombi in a stenotic pulmonary artery. D. Angioscopic images of the thrombi in the left common iliac vein. An arrow indicates the adhered pink surface.

Ultrasonography revealed deep vein thrombosis on both sides of the soleal vein. The early mortality risk was intermediate-high. Her blood pressure decreased to 87/68 mmHg, and the simplified pulmonary embolism severity index (sPESI) score became two. As hemodynamics seemed to become unstable, catheter-directed treatment was chosen. Emergent invasive right pulmonary arteriography revealed a defect, indicating thrombotic occlusion of A^2^, A^3^, A^4^, A^5^, and A^6^ in the right pulmonary artery (Figure 2A) and A^3 ^in the left and pulmonary artery (Figure 2B). Pulmonary artery pressure was 40/22 mmHg. NOGA-guided selective pulmonary thrombectomy was performed. Angioscopic images were obtained using the VISIBLE Fiber imaging system (FT-203F, Fiber Tech Co. Ltd., Tokyo, Japan) and a standard console (Intertec Medicals Co. Ltd., Osaka, Japan). NOGA showed mixed emboli in the reddish intima of the pulmonary artery (Figure 2C) and the common iliac vein (Figure 2D).

**Figure 3:**
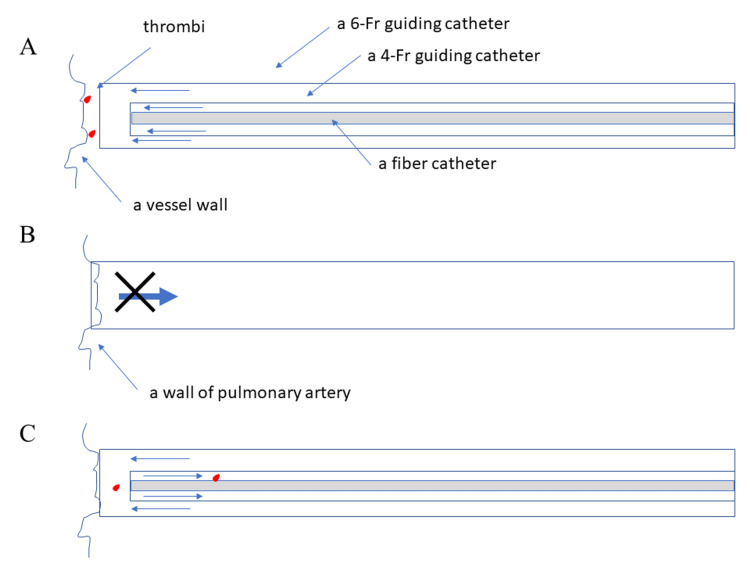
Schemas of angioscopic system A. A schema of the dual infusion system of non-obstructive angioscopy. Low-molecular-weight dextran was infused from 4-Fr and 6-Fr catheters, and the visual field was obtained. B. A schema of a catheter that adhered to a vessel wall, under low-pressure aspiration. C. A schema of the original method to aspirate under low pressure. Aspirating using a 6-Fr catheter while infusing low-molecular-weight dextran from a 4-Fr catheter.

Aspiration was performed using the NOGA system. The low-molecular-weight dextran NOGA system is a double-guide system using 4-Fr and 6-Fr catheters. In the NOGA system, low-molecular-weight dextran is infused manually through 4-Fr and 6-Fr catheters to obtain a visual field (Figure 3A) [12]. The massive thrombi were aspirated using 6-Fr catheters (Figure 3B) [13]. The catheter adheres to the vessel wall, and aspirating small fragmented thrombi may be stopped due to negative pressure inside the catheter because of the low pressure in the pulmonary artery (Figure 3B). A 6-Fr catheter was successfully used for aspiration, using an infusion of low-molecular-weight dextran, from a 4-Fr catheter (aspiration with single infusion method; Figure 3C).

**Figure 4:**
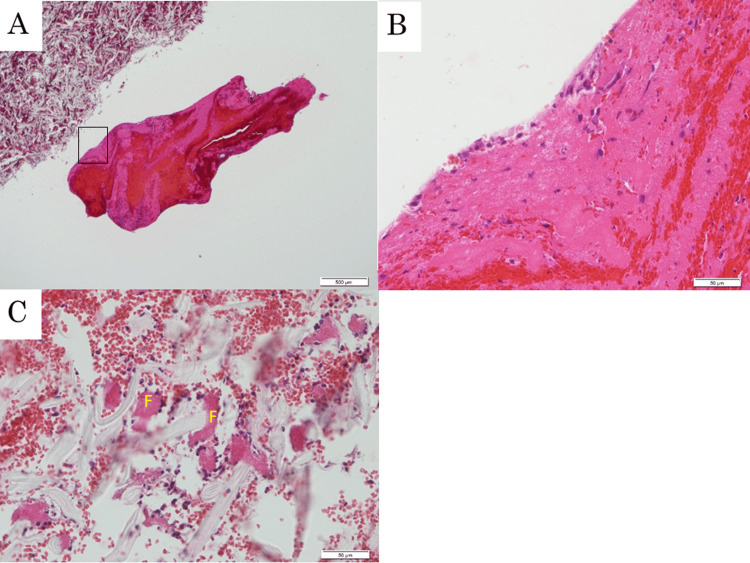
Hematoxylin-eosin-stained histopathology A. The fresh massive thrombi. Bar: 500 μm. B. A gross image of the area surrounded by a rectangle in Figure 4A. Bar: 50 μm. C. The fragments of the fresh thrombi. Bar: 50 μm. F, fragments of thrombi.

After aspirating the thrombi using the original method, massive thrombi (Figures 4A, 4B) and thrombus fragments (Figure 4C) were aspirated effectively.

In addition, 13,750 IU/body weight monteplase (Eisai Co., Ltd., Tokyo, Japan) was infused intravenously. Thrombi in the A^6^ segment of the right pulmonary artery was monitored for 30 min using NOGA. The red or pink-colored thrombi were detached from the wall of the pulmonary artery two minutes after infusion. The thrombi lost their erythematous color and dissolved partially six minutes after infusion. Meanwhile, the white thrombi gradually dissolved (Video 1).

**Video 1:** Time-series angioscopy video of A3 before thrombolysis (left) and 2, 4, 6, 8, and 10 minutes after thrombolysis (right). Time-series angioscopy video of A^3^ before thrombolysis (left) and 2, 4, 6, 8, and 10 minutes after thrombolysis (right). A video was recorded at baseline for a comprehensive analysis. The erythematous or pink thrombi from the pulmonary artery wall were detached two minutes after the infusion. The thrombi lost their erythematous color and were partially dissolved six minutes after the infusion. The white thrombi gradually dissolved.

The thrombi floated and disappeared 30 min after infusion of monteplase (Video 2).

**Video 2:** Angioscopy video of A3 at 30 minutes after thrombolysis The thrombi disappeared, and the erythematous intima was observed.

**Figure 5:**
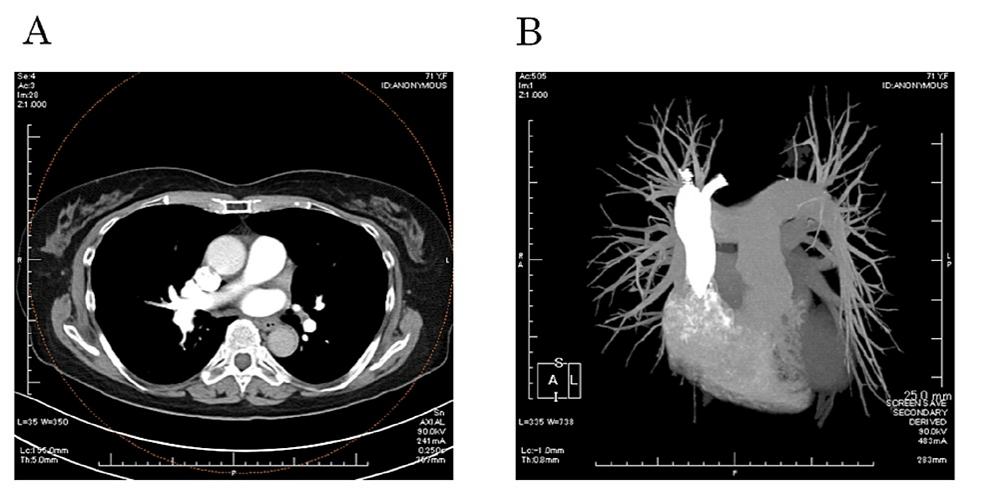
Computed tomography angiography of the pulmonary arteries on day 9. The thrombi were resolved. A. An axial image. Thrombi were resolved compared with that at baseline. B. Three-dimensional computed tomography angiography did now show a thrombus in the pulmonary arteries.

SpO_2_ increased to 98% on room air, and her dyspnea resolved. Edoxaban 60 mg was administered orally, and the thrombi rapidly resolved on computed tomography angioscopy after nine days (Figures 5A, 5B).

The D-dimer level was 2.0 μg/mL for 14 days. Ultrasonography and computed tomography did not point out vein compression or cancer. The patient was discharged with normal pulmonary pressure.

## Discussion

Proper diagnosis and prompt treatment are essential to reduce the mortality rate associated with PE. In six European countries with a total population of 454.4 million, more than 370 000 deaths were related to venous thromboembolism in 2004 [14]. Among these patients, 34% died suddenly or within a few hours after the event, before therapy was initiated or in effect. Among the remaining patients, 58% died due to acute PE, diagnosed postmortem, and only 7% of patients, who died early, were correctly diagnosed with PE before their death. The mortality rate associated with PE depends on the severity of the disease [15]. Compared to heparin monotherapy, thrombolytic therapy resulted in faster improvement of pulmonary obstruction and pulmonary pressure in patients with PE [16]. Mechanical reperfusion is used for mechanical fragmentation or thrombus aspiration. In the NOGA system, a clear liquid is injected into the catheter system to make the front of the fiber catheter transparent and obtain a clear view [12]. However, the pressure of the liquid from the catheter tip is much lower than the strong pressure of manual operation, and there is no risk of thrombus scattering downstream. If a thrombus being observed appears at risk of scattering downstream, it can be prevented by aspirating. The method shown in Figure 3C is a technique for washing and retrieving thrombi on the surface of the pulmonary artery. Since the surface of the catheter adheres closely to the vascular surface, any dislodged thrombi will not scatter within the vessel. Due to the complex structure, with high variations of the pulmonary artery, there has not been a catheter dedicated to the pulmonary artery until now. Although pulmonary arteriography was performed in advance to obtain anatomical information, attempts were made to minimize thrombi in any reached branch because navigating a catheter to a suspected branch with a thrombus was difficult. Migration of a catheter into small branches might cause pulmonary hemorrhage. Additionally, residual thrombi are one of the causes of chronic pulmonary hypertension. The Ikari (IL) catheter can reach any anatomy of pulmonary artery branches [17]. Furthermore, under NOGA guidance, the catheter can be advanced toward the thrombus and identify the type of thrombus for safe aspiration without damage to the pulmonary arteries. More commonly, it is integrated into a pharmacomechanical approach, combining mechanical or ultrasound fragmentation of the thrombus with in situ reduced-dose thrombolysis [1]. However, few modalities can demonstrate massive or fragmented thrombi to evaluate the effect of the intervention.

NOGA-guided selective aspiration thrombectomy was effectively performed to observe the features of a thrombus in a patient with PE [11]. Fresh thrombi are softer and easier to aspirate than chronic thrombi. Chronic thrombi are difficult to aspirate because their organization makes them solid. In the present case, the thrombi appeared fresh upon observation using NOGA. Different aspiration techniques were used, depending on the nature of the thrombi, particularly a mass or fragment. Catheter-based revascularization removes obstructive thrombi from the main or lobar pulmonary arteries, facilitates right ventricular recovery, improves symptoms, and reduces mortality and long-term complications [18]. However, the thrombus fragments densely adhered to the pulmonary artery wall. The thrombus fragments were insufficient targets for thrombectomy because the adherence of the catheter to the vessel wall during aspiration interrupts the removal of the thrombi while increasing the risk of vessel wall injury. Aspiration with circulating low-molecular-weight dextran inside the NOGA system effectively removed thrombus fragments without damaging the pulmonary artery wall. NOGA-monitored systemic thrombosis facilitates fibrinolysis in addition to hemodynamic changes. The duration for systemic thrombolysis to be effective remains unknown. NOGA patients experienced rapid thrombolysis within minutes after fibrinolysis therapy. Thrombus fragments, widely spread in the pulmonary arteries and cannot be aspirated, are observed on NOGA. Meanwhile, drug intervention is reduced. Fresh thrombi diminished more rapidly than organized thrombi. This reduced the risk of bleeding associated with catheter-directed low-dose thrombolysis [19].

Even with appropriate anticoagulant therapy, pulmonary artery pressure and right ventricular function may remain abnormal in 10-30% of patients, and 0.5-4% may develop chronic thromboembolic pulmonary hypertension (CTEPH), which is the most serious long-term complication of acute pulmonary embolism [20]. CTEPH may develop in the distant period even when thrombolytic therapy in the acute period is effective. Therefore, although NOGA showed that thrombolytic therapy was remarkably effective, it was only a remarkably effective treatment in the acute phase, and whether CTEPH develops is another matter. In preventing CTEPH, careful follow-ups, such as long-term pulmonary perfusion scintigraphy and pulmonary artery pressure measurement by right heart catheter, are also required. The patient has been followed carefully, and she has been free of CTEPH for three years.

## Conclusions

Few modalities can demonstrate massive or fragmented thrombi to evaluate the effect of thrombosis on PE. NOGA is a technique that visualizes the interior of the vessels, regardless of the vessel diameter. The combination of NOGA-guided selective pulmonary thrombectomy and NOGA-monitored systemic thrombolysis resulted in the patient’s rapid recovery with PE. More clinical cases are necessary to determine the safe dosages of thrombolysis.
